# A newly designed disk-lobe occluder with isogenous barbs for left atrial appendage closure: Initial multicenter experience

**DOI:** 10.3389/fcvm.2022.974994

**Published:** 2022-09-02

**Authors:** Yuan Bai, Xuechao Tang, Xudong Xu, Xianxian Zhao, Yawei Xu, Wei Chen, Xianyang Zhu, Qiguang Wang, Zhihua Han, Changqian Wang, Lu He, Yushun Zhang, Xin Pan, Cheng Wang, Lianglong Chen, Xuejiang Cen, Baiming Qu, Ni Zhu, Sha Zhang, Xinmiao Huang, Yongwen Qin

**Affiliations:** ^1^Department of Cardiology, Shanghai Changhai Hospital, Navy Military Medical University, Shanghai, China; ^2^Department of Cardiology, The 960th Hospital of People’s Liberation Army, Tai’an, China; ^3^Department of Cardiology, Shanghai Tenth People’s Hospital, Tongji University School of Medicine, Shanghai, China; ^4^Department of Congenital Heart Disease, General Hospital of Northern Theater Command, Shenyang, China; ^5^Department of Cardiology, Shanghai Ninth People’s Hospital, Shanghai Jiao Tong University School of Medicine, Shanghai, China; ^6^Department of Structural Heart Disease, The First Affiliated Hospital of Xi’an Jiaotong University, Xi’an, China; ^7^Department of Cardiology, Shanghai Chest Hospital, Shanghai Jiao Tong University, Shanghai, China; ^8^Department of Cardiology, Fujian Medical University Affiliated Union Hospital, Fuzhou, China; ^9^Department of Cardiology, Zhejiang Provincial People’s Hospital, Hangzhou, China

**Keywords:** stroke, atrial fibrillation, left atrial appendage, percutaneous left atrial appendage closure, complications

## Abstract

**Background:**

Although the implant success rate of left atrial appendage closure (LAAC) has increased and complications have decreased over time, there are still anatomically and technically complicated cases where novel LAA occluders may simplify the procedure and thus might potentially improve the clinical outcome.

**Objectives:**

This study aimed to assess the safety and efficacy of the newly designed device with isogenous barbs in LAAC.

**Methods:**

Eight centers in China participated in this prospective study from July 2016 to April 2018. Peri- and post-procedural safety and efficacy were evaluated through scheduled follow-ups and transesophageal echocardiography (TEE).

**Results:**

A total of 175 patients with a mean age of 68.4 ± 9.2 years old, a mean CHA_2_DS_2_-VASc score of 4.7 ± 1.8, and a mean HAS-BLED score of 3.2 ± 1.3, were included. The device was successfully implanted in 173 patients (98.9%). The device size ranged from 18 to 34 mm. Clinically relevant pericardial effusion (PEF) in the perioperative period, occurred in 3 patients (1.7%). TEE follow-up was available in 167 (96.5%) patients at 12-month. During follow-up, 9 patients suffered serious adverse event: 4 death (2.3%), 1 ischemic stroke (0.6%), and 2 gastro-intestinal bleeding (1.2%) and 2 device-related thrombus (DRT) (1.2%). Estimated annual thromboembolism rate reduced by 90% and estimated annual major bleeding rate reduced by 81% after LAAC with the newly designed device.

**Conclusion:**

The newly designed device with isogenous barbs for LAAC could be performed effectively with a low incidence of adverse events and a high incidence of anatomic closure.

## Introduction

Percutaneous left atrial appendage closure (LAAC) has been developed as an alternative treatment for the prevention of stroke in high-risk patients with non-valvular atrial fibrillation (NVAF) and contraindication to chronic oral anticoagulant therapy (1). Both clinical trials and real-world studies have confirmed that LAAC plays a positive role in preventing stroke and reducing bleeding in NVAF patients (1–4). There are two types of left atrial appendage (LAA) occluder in clinical practice: single-seal device and dual-seal device. Clinically relevant pericardial effusion (PEF) is one of the most frequent and severe procedure-related complications after LAAC, which is closely related to implanters’ experience and barbs of occluder. The incidence of clinically relevant PEF in the initial experience with the dual-seal mechanism first-generation Amplatzer Cardiac Plug (ACP; St. Jude Medical, United States) device was as high as 3.5% (5). In order to improve the safety of LAAC with dual-seal device, our center designed and developed the LACbes^®^ (PushMed, Shanghai, China) device with isogenous barbs. This prospective, multicenter clinical study reported the initial assessment of the feasibility and safety of the LACbes^®^ device in patients with NVAF.

## Materials and methods

### Study design

The present study was a prospective, non-randomized, multi-center study. Enrollment was started in July 2016 and finished in April 2018. The study protocol conforms to the ethical guidelines of the 1975 Declaration of Helsinki. Informed consent was obtained from each patient.

### Study population

Inclusive criteria: (1) CHADS_2_ ≥ 1; (2) HAS-BLED ≥ 2; (3) high risk or history of falls; (4) patients at high-risk of bleeding; (5) patient preference. Exclusion criteria included patients with thrombus formation in the left atrium (LA) or LAA, endocarditis, sepsis, ejection fraction < 30%, and contraindications to antiplatelet therapy. Detailed exclusion criteria are shown in Supplementary Table 1.

### LACbes^®^ system characteristics

**FIGURE 1:**
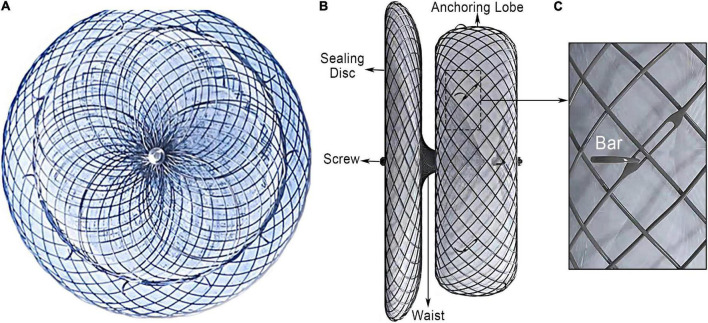
LACbes^®^ left atrial appendage device. **(A)** Side view of left atrial appendage; **(B)** lateral view of the occluder; **(C)** isogenous barb.

The LACbes^®^ occluder is a self-expanding device constructed from a nitinol mesh, and it consists of a distal anchoring lobe and a proximal sealing disc, which are connected by an articulated waist (Figures 1A,B) (6). The device is designed according to the “anchor and seal” principle, with the lobe anchoring in the LAA and the disc sealing the LAA ostium. The proximal sealing disc is arched attached by end-screw and filled with polyester patches. The distal anchoring lobe is a flat cylinder filled with sewn in polyester patches. Unique features of the device include isogenous barbs, a flexible waist, and a malleable sealing disc. Ten to twelve isogenous barbs are placed in the anchoring lobe circumferentially (Figure 1B). Barbs are curve-shaped, with distal end facing to the anchoring lobe. The head of the barbs received passivation treatment, and the side edge of the barbs is square-flat (Figure 1C). According to *in vitro* experiment, the LACbes^®^ occluder can be retrieved and deployed repeatedly without deforming the barbs for 100 times (Supplementary Video 1). The available anchoring lobe sizes of device range from 18 to 34 mm (with a size increment of 2 mm). The sealing disc is 6–10 mm larger in diameter than the anchoring lobe. The dedicated delivery sheath has two 45^°^ out- of-plane bends so that the axial direction of the sheath canal is consistent with that of the LAA.

### Percutaneous closure of left atrial appendage procedure

The procedure was performed under general/local anesthesia and transesophageal echocardiography (TEE) guidance, *via* femoral vein approach. The LA was accessed through transseptal puncture, a patent foramen ovale or an atrial septal defect (7, 8). If the PFO is tunnel-shaped, through which it may be difficult to properly intubate the LAA with the delivery sheath, access through transseptal puncture is recommended. Heparin is then administered to maintain the ACT > 250 s for the duration of the procedure. After LAAC, the patent foramen ovale or atrial septal defect was closed at the same sitting. An angiography in 30°right anterior oblique + 20°caudal was performed through a 5F pigtail catheter placed in the LAA. The largest angiographic and TEE (0°/45°/90°/135°views) diameter of the LAA ostium and landing zone were measured, respectively. According to manufacturer instructions, an inner diameter of the anchoring lobe of the LACbes^®^ device, which was 3–5 mm larger than that of the landing zone, was typically adopted. After the device was deployed, a tug test was carried out to confirm the safe placement before its final release. Correct positioning of the device at the LAA ostium must be confirmed by TEE and angiography. Procedural success was defined as device implantation in correct place. Performing an angiogram through the sheath with only the anchoring lobe deployed can be quite helpful to ensure proper lobe orientation When properly positioned, the LACbes^®^ device will fulfill “PAST” criteria for release, which is (1) Proper Position: the anchoring lobe is positioned at the landing zone 2/3rd below the left circumflex; (2) Absolute Anchor: tug the curve sealing disc to cage shape, the anchoring lobe was still fixed in the LAA landing zone without movement; (3) Separate Seal: the sealing disc is set apart from the anchoring lobe; the sealing disc covers the orifice with no or only a small amount of residual shunt; (4) Typical Tire: anchoring lobe should be a shape of tire.

### Antithrombotic therapy and follow up

Patients who received successful device implantation were discharged on aspirin (100 mg) and clopidogrel (75 mg) for at least 180 days. Patients were followed with electrocardiography, transthoracic echocardiography (TTE), TEE and clinical examinations immediately, 3 and 12 months after procedure. Additionally, TEE or TTE also was performed at other times at the discretion of the investigator, if clinically indicated. There was an independent Echo core Lab for TEE analysis. If TEE criteria for successful sealing (peri-device leak < 3 mm) of the LAA were fulfilled at 90 days, dual-antiplatelet therapy was continued until 6 months, followed by lifelong aspirin or clopidogrel alone. If a peri-device leak (PDL) larger than 3 mm was measured at 90-day, patients continued dual-antiplatelet therapy until PDL was smaller than 3 mm. If DRT was observed on TEE follow-up, antithrombotic therapy was adjusted to oral anticoagulation until complete thrombus resolution. Once there is a bleeding event, the antithrombotic regimen was adjusted according to the operators’ discretion.

### Major adverse events

Major adverse events included death, myocardial infarction, stroke/transient ischemic attacks (TIA), systemic embolism, device embolization, clinically relevant PEF, device-related thrombus (DRT), PDL, and major bleeding. Clinically relevant PEF was defined as requiring pericardiocentesis, surgical intervention or blood transfusion, or resulting in shock and/or death (9). Major bleeding was defined as fatal or major with hemoglobin drop of 3 g/dL, requirement of packed red blood cell transfusions or intracranial hemorrhage (10). Leaks were categorized according to the width of the color jet on TEE as follows: trivial (< 1 mm), mild (1–3 mm), or significant (> 3 mm). Minor adverse events included clinically non-relevant PEF, vascular complication (including pseudoaneurysm, arteriovenous fistula and femoral hematoma) and minor bleeding. The primary endpoint was device efficacy to prevent stroke, TIA, and systemic embolism and the secondary endpoints included the incidence of death, major bleeding, PEF, PDL, and DRT.

### Statistics

Descriptive data for continuous variables were presented as mean ± *SD*. Categorical variables were presented as relative frequencies. Given the descriptive nature of this study and small sample size, no between-group comparison was performed. All statistical analyses were performed with commercially available software (PASW Statisticsv20.0.0; SPSS, Inc., Chicago, IL).

## Results

### Baseline characteristics

There were 175 patients enrolled in the study in 8 cardiology centers in China. Their baseline characteristics were shown in Table 1. The mean patient age was 68.4 ± 9.2 years and 53.7% were men. A total of 133 patients (75.9%) had a history of previous stroke or TIA. The average CHA_2_DS_2_-VASc score was 4.7, representing a 5.8% annual risk for stroke (11); while the average HAS-BLED score was 3.2, representing a 6.4% annual risk for major bleeding (12).

**TABLE 1:** Baseline demographic characteristics.

Variable	
Age, years	68.4 ± 9.2
Age ≥ 75, years	42/175 (24.0%)
Male (%)	94/175 (53.7%)
Ischemic stroke (%)	72/175 (41.1%)
Hemorrhagic stroke (%)	10/175 (5.7%)
TIA (%)	51/175 (29.1%)
**NYHA class (%)**	
I	18/175 (10.3%)
II	126/175 (72.0%)
III	31/175 (17.7%)
IV	0/175 (0.0%)
LVEF,%	62.5 ± 5.5
CHA_2_DS_2_ score	3.0 ± 1.3
HAS-BLED score	3.2 ± 1.3
CHA_2_DS_2_-VASc score	4.7 ± 1.8
Hypertension (%)	132/175 (75.4%)
Diabetes mellitus	37/175 (21.1%)
Major bleeding	32/175 (18.3%)
Systemic embolism	5/175 (2.9%)
Heart failure	81/175 (46.3%)
**LAA morphologies**	
Chicken wing	39/175 (22.3%)
Windsock	30/175 (17.1%)
Cactus	61/175 (34.9%)
Cauliflower	45/175 (25.7%)

TIA, Transient Ischemic Attacks; LVEF, Left Ventricular Ejection Fraction.

### Procedural results

**FIGURE 2:**
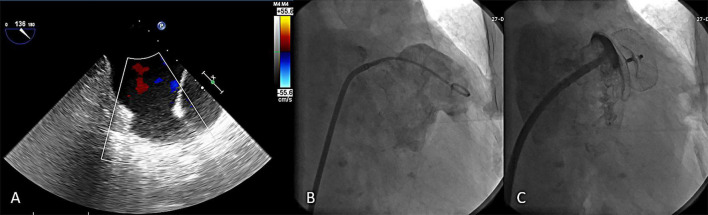
The largest LACbes device (34 mm) in a shallow left atrial appendage. **(A)** TEE in 135° view showed a large and shallow LAA; **(B)** LAA angiography in a RAO 30° + caudal 20° projections showed a “chicken wing” LAA; **(C)** good sealing after LACbes device implantation under LAA angiography. LAA, left atrial appendage; TEE, transesophageal echocardiography.

The device was successfully implanted in 173 patients (98.9%). Two subjects had unsuitable LAA anatomy for the LACbes^®^ device implantation and two devices were removed due PEF. Four patients (2.3%) presented a PDL larger than 3 mm after releasing the LACbes^®^ occluder. The maximum width of the leak measured was 4.3 mm. The device size ranged from 18 to 34 mm (Table 2). The largest anchoring lobe size of LACbes^®^ was 34 mm. In that patient, TEE in 135° view showed a large and shallow LAA (Figure 2A). LAA angiography showed a “chicken wing” LAA with a maximal diameter of 31 mm and depth of 20 mm (Figure 2B). Finally, the patient underwent a successful LAAC without PDL (Figure 2C). Clinically relevant PEF, the only major complication in the perioperative period, occurred in 3 patients (1.7%) (Table 2). Among them, one patient was treated with pericardiocentesis, and the symptoms were significantly relieved. The remaining two patients were treated with surgical intervention after pericardiocentesis due to continuous bleeding. One patient was treated with device removal and perforation ligation. In the other patient, the occluder was removed and the LAA was resected due to badly damaged. Besides, there was one case of self-limited mild PEF 3 days after LAAC. All the patients with PEF discharged in good condition. No episodes of deaths, stroke, systemic embolism, TIA, vascular complication, major bleeding, or device embolization occurred in the periprocedural period.

**TABLE 2:** Procedural details and clinical outcomes at follow-up.

Variable	
**Procedural details**	
Procedural success (%)	173/175 (98.9%)
**Device size (mm)**	
18	7/173 (4.0%)
20	11/173 (6.4%)
22	36/173 (20.8%)
24	37/173 (21.4%)
26	40/173 (23.1%)
28	20/173 (11.6%)
30	12/173 (6.9%)
32	8/173 (4.6%)
34	2/173 (1.2%)
Death	0/175 (0%)
Clinically non-relevant PEF (%)	1/175 (0.6%)
Clinically relevant PEF (%)	3/175 (1.7%)
Stroke/TIA	0/175 (0%)
Major bleeding	0/175 (0%)
Device embolization	0/175 (0%)
Myocardial infarction	0/175 (0%)
Vascular complication	0/175 (0%)
LAA leak	
Residual flow < 1 mm	6/173 (3.5%)
Residual flow 1–3 mm	18/173 (1.0%)
Residual flow > 3 mm	4/173 (2.3%)
**Clinical outcomes during 12-month follow-up**	
Number (%)	173/175 (98.9%)
Death (%)	4/173 (2.3%)
Clinically non-relevant pericardial effusion (%)	1/173 (0.6%)
Ischemic stroke/TIA (%)	1/173 (0.6%)
Hemorrhagic stroke (%)	0/173 (0%)
Major bleeding (%)	2/173 (1.2%)
Systemic embolism	0/173 (0%)

PEF, pericardial effusion; TIA, Transient Ischemic Attack.

### Clinical and echocardiographic follow-up

Clinically follow-up was available in 173 patients (98.9%). A total of 10 patients (5.8%) suffered serious adverse event during 12-month follow-up, including 4 death (2.3%), 1 ischemic stroke (0.6%), 2 gastro-intestinal bleeding (1.2%), 2 DRT (1.2%), and 1 significant PDL (0.6%). Additionally, one patient presented with shortness of breath 5 weeks after LAAC. There was no tamponade physiology on TTE. The symptom was relieved and effusion disappeared gradually after oral administration of diuretics. A total of 4 patients died during the follow-up period. One patient had a sudden cardiac death 2 months after LAAC, without autopsy. One patient with Myelodysplastic syndrome was died of multiple organ dysfunction syndrome 9 months after LAAC. Another patient died 4 months after LAAC due to a traffic accident. The last cause of death was severe pneumonia based on chronic heart failure 1 month after LAAC.

**FIGURE 3:**
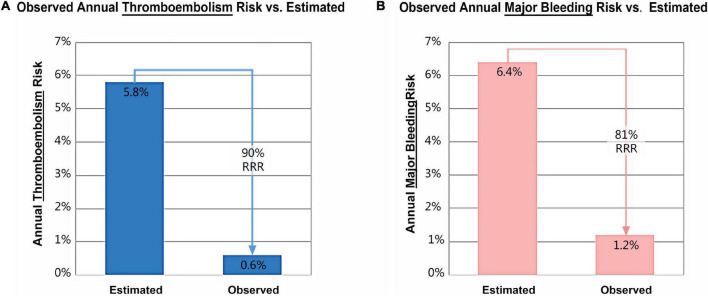
Relative risk reduction (RRR) in annual thromboembolism **(A)** and major bleeding **(B)** after left atrial appendage closure with LACbes^®^.

In addition, one patient developed dizziness 7 months after LAAC, diagnosed with ischemic stroke based on the history and radiographic data. Compared with an estimated annual thromboembolism rate of 5.8% based on CHA_2_DS_2_-VASC score, the observed rate was 0.6%, conferring an 90% relative risk reduction after LAAC (Figure 3A). A total of 5 patients suffered from bleeding during 12 months of follow-up, including 3 subconjunctival and 2 gastro-intestinal bleeding. The observed annualized major bleeding rate after LAAC was 1.2%, which was obviously lower than the expected bleeding rate (6.4%) based on HAS-BLED score (Figure 3B).

TEE follow-up at 3 and 12-month was available in 169 (96.6%) and 167 (95.4%) patients, respectively. The incidence of significant PDL decreased with time from 1.2% (2/169) at 3-month to 0.6% (1/167) at 12-month (Table 3). DRT was observed in two patients (1.2%) at the 1-year TEE follow-up. The two patients were treated with 3.5-month oral anticoagulation until complete thrombus resolution. No neurological and cardiovascular events were observed among patients with PDL and DRT during 12-month follow-up.

**TABLE 3:** TEE examination during follow-up.

Variable	3 months	12 months
Number (%)	169/175 (96.6%)	167/175 (95.4%)
**PDL**		
Residual flow < 1 mm (%)	10/169 (5.9%)	6/167 (3.6%)
Residual flow 1–3 mm (%)	8/169 (4.7%)	2/167 (1.2%)
Residual flow > 3 mm (%)	2/169 (1.2%)	1/167 (0.6%)
Device-related thrombus (%)	0/169 (0%)	2/167 (1.2%)

PDL, peri-device leak.

## Discussion

This study is the first multicenter evaluation of percutaneous LAAC with the LACbes^®^ system in patients with NVAF. The device was successfully implanted in 98.9% of patients with a low rate of major complications (1.7%) in the perioperative period and serious adverse event (5.8%) during follow-up. Estimated annual thromboembolism rate reduced by 90% and estimated annual major bleeding rate reduced by 81% after LAAC with the LACbes^®^ device.

The data of PRAGUE-17 revealed that LAAC was non-inferior to new oral anticoagulants for preventing stroke with significantly reducing non-procedural bleeding (13). This research further confirms that LAAC can be used as an alternative to long-term anticoagulant therapy in patients with NVAF. At present, there are two types of LAA occluders in clinical practice: single-seal device, such as Watchman (Boston Scientific, United States) and Watchman-FLX (Boston Scientific, United States), and dual-seal device, such as ACP and Amplatzer Amulet (Abbott, United States). In Kleinecke’s study, the overall rate of major peri-procedural complications was 4.1% in the Watchman and 6.0% in the ACP group (14). In the Amulet IDE trial, the rate of major procedure-related complications with the Amulet device was nearly twice higher than that with the Watchman 2.5 device (4.5% vs. 2.5%) (15). In the SWISS-APERO trial, the incidence of major procedure related complications in the Amulet group was higher than that in the Watchman 2.5/Watchman FLX (9.0% vs. 2.7%) (16). Thus, the LAAC procedure with dual-seal device seems less safe than that with single-seal device. However, the Belgian LAAO Registry and the multicenter FLAAC registry showed the LAAC is effective and relatively safe in a real-world setting, using either the WATCHMAN or the ACP/Amulet device (17, 18). As a novel dual-seal device with isogenous barbs, the LACbes^®^ device showed a lower incidence (1.7%) of major peri-procedural complications.

The procedural success rate of LAAC with the ACP and Amulet device 96.0–97.3% and 94.6–98.4%, respectively (5, 14–16, 19, 20). In this study with the LACbes^®^ system, the procedural success rate was 98.9%, which was similar to that rate of the ACP and Amulet device. Such a high technical success rate attributes to the unique structure of the LACbes^®^ device. First, its waist can be extended a little and even bend at an angle to self-orient to the cardiac wall and make the occluder more adjustable and flexible during implantation. Moreover, the nitinol filaments are thinner in the nitinol mesh of the sealing disc than that of the anchoring lobe, which makes the LACbes^®^ device more suitable to different morphological ostium of LAA.

Clinically relevant PEF is one of the most severe complications after LAAC. The rate of clinically relevant PEF of LAAC in the perioperative period with ACP and Amulet was 3.5–3.8%, 2.7–4.0%, respectively (5, 14–16, 19). In the multicenter experience with ACP, the incidence of clinically relevant PEF was relatively low (1.4%), yet two patients died of PEF (20). Two possible mechanisms may account for the lower PEF rate of the LACbes^®^ system (1.7%) compared with that of the ACP and Amulet device. First, the head of the barbs received passivation treatment, which was atraumatic to LAA and reduced the risk of the barbs puncturing LAA. Second, if barbs caused a little perforation in the LAA, the membrane in the anchoring lobe would cover the hole immediately and clinically relevant PEF would be avoided.

The independent predictors of PEF after LAAC includes advanced age, higher CHA_2_DS_2_-VASc score, and obesity (21). The common cause of PEF within 7 days of the procedure has generally been considered to be cardiac perforation due to improper manipulation of transseptal puncture, delivery system or the closure device. Common causes for delayed PEF include delayed LA or LAA perforation due to penetration of the barbs of occluder and heart failure. Xiao’s study revealed that an umbrella without fully opening was associated with delayed PEF events after LAAC (22). For the patient in our study, the symptoms of PEF were significantly relieved after diuretic treatment and pericardiocentesis was avoided. Therefore, we speculate that in addition to the chronic injury of metal barbs, heart failure may also be an important factor of delay PEF.

Device embolization is a rare and severe complication after LAAC. The incidence of device embolization in LAAC with ACP and Amulet was 0.8–3.0% and 0.7–2% (5, 14–16, 19, 20). In our small series study, no device embolization occurred, which was attributed to multiple isogenous barbs incorporated into the circumference of the distal anchoring lobe to stabilize the device in LAA. Besides, a surface-to-surface contact between the side edge of barbs and the wall of LAA was incorporated, making the stress distribution in the occluder evenly and its fixation firm. Additionally, the LACbes^®^ device can be advanced distally before final release due to the closed atraumatic distal end and application of the new “ball technique,” which helps to position device not only stably but also safely.

The LAA shape and size varies from person to person, and PDL is common following device implantation. The incidence of significant PDL after LAAC with Watchman was 11.8% at 12-month follow-up (23). In the multicenter experience with ACP, the incidence of significant PDL was 1.9% at 7-month follow-up. In the Amulet IDE trial, the incidence of PDL > 3 mm in the Watchman group 45 days post LAAC was significantly higher than that in the Amulet group (25% vs. 10%). In this study, the incidence of significant flow around the occluder was 1.2% at 3-month and 0.6% at 12-month. The reason why the PDL rate seems lower in the dual-seal device may be that TEE is not sensitive in evaluating leak around the dual-seal device. Cardiac CT angiography (CCTA) appears to be a much more sensitive modality to detect residual leak compared with TEE. In 2014, Saw et al. found that all occluded LAA have an attenuation of < 100 Hounsfield unit and < 25% of the contrast opacification of the LA on CCTA (24). Based on above finding, the degree of LAA patency was classified as non-patent, PDL, intra-device leak, mixed leak, and patent appendage with no visible leak (16). In this way, Amulet was not superior to Watchman in terms of LAA patency at 45-day CCTA in the SWISS-APERO trial (16). In the future, the concept of LAA patency can be applied clinically to evaluate the sealing capacity of the occluder after LAAC.

The study is a non-randomized and observational trial. Furthermore, there are still other limitations, such as small sample size, relatively short follow-up, and lack of head-to-head comparison with other devices for LAAC. Therefore, it is not sufficient to conclude the long-term efficacy and safety of the LACbes^®^ device in stroke prevention for NVAF patients. Selection biases might also be present due to a very low rate of hemorrhagic stroke patients included. Larger, randomized, controlled (LACbes^®^ vs. oral anticoagulant agents) clinical trials with extended follow-up are needed in the future to assess the safety and efficacy of this novel and promising percutaneous system for LAA occlusion.

## Conclusion

Our preliminary data showed implantation of the LACbes^®^ device was a generally safe and feasible method for percutaneously sealing the LAA with low stroke and major bleeding risk. Furthermore, LACbes^®^ was suitable for different morphological ostium of LAA, easy to implant, stable in the LAA, and less traumatic to the wall of LAA.

## Data availability statement

The raw data supporting the conclusions of this article will be made available by the authors, without undue reservation.

## Ethics statement

The studies involving human participants were reviewed and approved by the Changhai Hospital Ethics Committee. The patients/participants provided their written informed consent to participate in this study.

## Author contributions

XXZ, YX, XYZ, CQW, YZ, XP, LC, BQ, and YQ: concept and design. SZ: data collection and statistics. XH and YB: funding. WC, QW, ZH, LH, YZ, CW, XC, and NZ: data analysis/interpretation and perform the LAAC procedure. YB and SZ: drafting of the manuscript. XH, NZ, and XXZ: critical revision of the manuscript for important intellectual content. YB and YQ: final approval of the manuscript submitted. All authors contributed to the article and approved the submitted version.

## Supplementary material

Supplementary Table 1 Detailed exclusion criteria.

Supplementary Video 1 LACbes can be retrieved and deployed repeatedly without deforming the barbs.
