# Supplementary material for: A newly designed disk-lobe occluder with isogenous barbs for left atrial appendage closure: Initial multicenter experience
Source: Front Cardiovasc Med. 2022 Sep 2;9:974994. doi: 10.3389/fcvm.2022.974994 (PMC9478548; doi:10.3389/fcvm.2022.974994)
Supplement: Supplementary Table 1 — Detailed exclusion criteria. [file Table_1.DOCX]

| Exclusion Criteria |
| --- |
| 1. Requires long-term oral anticoagulation therapy for a condition other than atrial fibrillation  2. Contraindicated for or allergic to aspirin, clopidogrel, or warfarin use  3. Indicated for chronic P2Y12 platelet therapy inhibitor  4. Is considered at high risk for general anesthesia  5. Stroke or transient ischemic attack (TIA) within 30 days prior to implant procedure  6. Myocardial infarction (MI) within 90 days prior to implant procedure  7. New York Heart Association Class IV Congestive Heart Failure  8. Left ventricular ejection Fraction (LVEF) <30%  9 Left atrial appendage is obliterated or surgically ligated  10. Hypersensitivity to any portion of the device material (e.g. nickel allergy)  11. Subject is pregnant or pregnancy is planned during the course of the investigation  12. Active endocarditis or other infection producing bacteremia  13. Subject whose life expectancy is less than 2 years  14. Presence of other anatomic or comorbid conditions, or other medical, social, or psychological conditions that, in the investigator’s opinion, could limit the subject’s ability to participate in the clinical trial or to comply with follow up requirements  15. thrombus formation in the left atrium  16. Significant mitral valve stenosis (i.e. mitral valve area <1.5 cm2 |

Table S1 Detailed exclusion criteria
